# Long non-coding RNA AFAP1-AS1 is a novel biomarker in various cancers: a systematic review and meta-analysis based on the literature and GEO datasets

**DOI:** 10.18632/oncotarget.21830

**Published:** 2017-10-11

**Authors:** Yumin Wang, Yongzhen Mo, Xiang Yang, Ruoyu Zhou, Zeyu Wu, Yuchen He, Xue Yang, Yaxian Zhong, Yajun Du, Hang Zhou, Xiaoling Li, Yong Li, Guiyuan Li, Zhaoyang Zeng, Can Guo, Wei Xiong

**Affiliations:** ^1^ Key Laboratory of Carcinogenesis of Ministry of Health, Xiangya Hospital, Central South University, Changsha, Hunan, China; ^2^ Key Laboratory of Carcinogenesis and Cancer Invasion of Ministry of Education, Cancer Research Institute, Central South University, Changsha, Hunan, China; ^3^ Hunan Key Laboratory of Nonresolving Inflammation and Cancer, Disease Genome Research Center, The Third Xiangya Hospital, Central South University, Changsha, Hunan, China; ^4^ Department of Cancer Biology, Lerner Research Institute, Cleveland Clinic, Cleveland, Ohio, USA

**Keywords:** long noncoding RNA, cancer, AFAP1-AS1, meta-analysis, biomarker

## Abstract

**Background:**

Growing evidence indicates that AFAP1-AS1 plays an important role in various cancers, suggesting that it might be a potential cancer biomarker.

**Materials and Methods:**

A meta-analysis was performed using microarray data obtained via the Affymetrix Human Genome U133 Plus 2.0 platform (found in the GEO database) and data obtained through a systematic search of PubMed and Web of Science. The pooled odds ratio (OR) and hazard ratio (HR) with 95% CI (confidence interval) were used to judge the value of biomarkers.

**Results:**

A total of 30 studies were included in this meta-analysis, comprising a total of 3573 patients. AFAP1-AS1 was significantly linked with overall survival (OS) (HR = 1.58; 95% CI: 1.12–2.23) and recurrence-free survival (RFS) (HR = 2.32, 95% CI: 1.68–3.19). We found that AFAP1-AS1 was a risk factor in the prognoses of lung cancer (pooled HR: 1.54; 95% CI: 1.01–2.34), digestive system cancer (pooled HR: 1.87; 95% CI: 1.45–2.41) and nasopharyngeal carcinoma (HR: 11.82; 95% CI: 5.09–27.46). AFAP1-AS1 was also a risk factor for RFS in breast cancer (pooled HR = 2.90; 95% CI: 1.69–4.98), as well as TNM stage in both esophageal cancer (pooled OR = 1.90; 95% CI: 1.01–3.57) and colorectal cancer (OR = 6.72; 95% CI: 1.92–23.58). AFAP1-AS1 was significantly associated with lymph node metastasis in clear cell carcinoma (OR = 5.04; 95% CI: 2.36–10.78) and distant metastasis in pancreatic cancer (OR = 11.64; 95% CI: 2.13–63.78).

**Conclusions:**

AFAP1-AS1 can serve as a novel molecular marker predicting tumor progression, patient prognosis and lymph node metastasis in different types of cancers.

## INTRODUCTION

Currently, cancers are a great challenge in the field of human health. Epidemiological data show that the global incidence rate for cancer amounts to approximately 14,000,000 per year and that mortality exceeds 8,200,000 [1, 2]. Early detection and diagnosis are critical for improving survival time and quality of life [3]. However, current clinical findings are mainly based on imaging analyses and are restricted by factors such as resolution. Additionally, tumors often cannot be diagnosed early [4, 5]. Fortunately, owing to their inherent sensitivity, tumor markers (especially molecular biomarkers) have revealed great potential in the early diagnosis of malignant tumors [6].

Long noncoding RNAs (lncRNA) are a type of noncoding RNA greater than 200 nucleotides in length [7]. In recent years, lncRNAs have been shown to play an important regulatory role in chromatin modification, X chromosome inactivation and transcription, translation, genetic imprinting, dosage compensation, and the regulation of protein activity and RNA alternative splicing [8–12]. LncRNAs play an important role in tumor incidence and development [13]. A variety of lncRNAs can be used as molecular tumor markers. These markers, together with clinical data, show potential value in the diagnosis and treatment of malignant tumors.

AFAP1-AS1 is a type of lncRNA encoded by the antisense strand of the AFAP1 gene. Our group and other research groups have found that AFAP1-AS1 plays an important role in the regulation of tumor incidence and development by promoting tumor cell metastasis [14–21]. We discovered that AFAP1-AS1 is closely correlated with clinical data - including survival time and TNM staging - in various cancers, such as nasopharyngeal carcinoma [14, 15] and lung cancer [16]. This demonstrates the potential value of AFAP1-AS1 as a new tumor marker. However, due to the restricted sample size, the credibility of previous studies remains doubtful. Thus, our study combined microarray data from the GEO database with results from several published studies in order to systemically review the prognostic value of AFAP1-AS1 in cancers and to determine the molecular mechanisms involved in the regulation of AFAP1-AS1 in tumorigenesis and development.

## MATERIALS AND METHODS

### Literature searching

Tumor microarray datasets based on the Affymetrix Human Genome U133 Plus 2.0 platform were obtained through independent searches of the GEO database by 3 reseachers (Wang, Mo, and Yang). We also retrieved all relevant published literature from Pubmed and Web of Science. The literature search was limited to studies published in the English language prior to July 2017. To increase the totality of the search, both mesh-terms and free words were used. Search terms were: ‘AFAP1-AS1’, ‘metastasis-associated lung adenocarcinoma transcript 1’, ‘long intergenic noncoding RNA’ or ‘lncRNA’ or ‘noncoding RNA’, ‘cancer’ or ‘carcinoma’ or ‘neoplasm’ and ‘prognosis’ or ‘survival’.

### Literature selection

Results from the literature search were screened prior to analysis of datasets. Eligible studies met the following criteria: involved any type of human cancer; detected and analyzed AFAP1-AS1 expression in tissues; literature study involved the correlation between AFAP1-AS1 expression level and overall survival (OS) or disease free survival (DFS); literature provided survival curve or hazard ratio (HR) and the 95% confidence interval (CI). Literature was excluded based on the following criteria: experts’ opinions, letters, comments, case reports, reviews and meeting reports; the article was not found in full; survival rate data files were not presented in the literature; related survival data could not be gained from the literature; repeatedly published literature. When the same data subsets were published in more than one article, only the latest publication was included. All included studies were obtained as full text. Controversies regarding study selection were resolved via discussion with investigator Wang.

### Data extraction

This process was carried out independently by six researchers and consensus was reached among all researchers for each dataset. For the 18 Affymetrix Human Genome U133 Plus 2.0 platform microarray datasets available in the GEO database (where both AFAP1-AS1 expression and the corresponding survival data was available), the following data were extracted: OS, RFS, lymph node metastasis, TNM stage, survival outcome, and AFAP1-AS1 expression value. For studies selected from Pubmed and Web of Science, the following items were extracted: author, publishing date, nationalities of patient groupings, sample size, expression level of AFAP1-AS1, length of follow-up, method of survival analysis, OR value and CI, OS, DFS, HR value and CI, RFS, lymphocyte metastasis, TNM staging, and distant metastasis. Screened studies were compiled and checked according to standards for meta-analyses. We then built the database and applied lnHR and SelnHR as a combination index. In cases where only the survival curve was provided, Engauge Digitizer 4.1 was applied to extract relevant data from the survival curve. We then performed calculations according to the method of Jayne F Tierney et al.

### Quality assessment of the primary studies

Three researchers (Wang, Mo, and Yang) independently evaluated the quality of all selected studies according to the method of Steels et al. [22, 23] The final assessment is expressed as a percentage, where higher scores indicate higher literature quality.

### Statistical approach

(1) We used a single factor cox analysis to calculate the HR value and its 95% CI, OS, and RFS from the gene microarray data. We used GraphPad Prism 5 to draw Kaplan-Meier survival curves based on AFAP1-AS1 expression levels. A ≥ 1.5-fold difference was used as the cut-off value for differentially expressed lncRNA and the false discovery ratio (FDR) was < 0.05. (2) Review Manager 5.3 was used to analyze the obtained data based on clinical indices including OS, DFS, and RFS. HR values from literature studies that acquired OS, RFS, and DFS were combined and heterogeneity was determined to be significant when I^2^ > 50% [4, 24]. A random effect model was adopted and sub-analyses were made when heterogeneity existed among study results. Otherwise, a fixed effect model was adopted to merge HR values and 95% CIs. Forest plots were applied to present calculation results. HR was calculated as the ratio of the prognoses in high AFAP1-AS1 cases to low AFAP1-AS1 cases. An HR > 1 demonstrated a poorer prognosis for the high AFAP1-AS1 expression group compared with the low AFAP1-AS1 expression group. (3) Publication biases were described through funnel plots for accessible OS and RFS data analysis. SelnHR was shown on the x-axis while lnHR was shown on the y-axis. Symmetry of the funnel plot was tested by linear regression models (Begge method and Eggs method) in STATA 12.0 to evaluate publication bias. Next, a sensitivity analysis was performed using STATA 12.0 to evaluate HR values for AFAP1-AS1 and OS extracted from single studies. Furthermore, OR values were combined and heterogeneity of clinical tumor diagnosis index data was analyzed using Review Manager 5.3. High AFAP1-AS1 expression was analyzed for correlation with TNM staging, lymphocyte metastasis, and distant metastasis across various cancer types. *P* < 0.05 indicated statistical significance of results.

## RESULTS

### Included studies and characteristics

**Figure 1:**
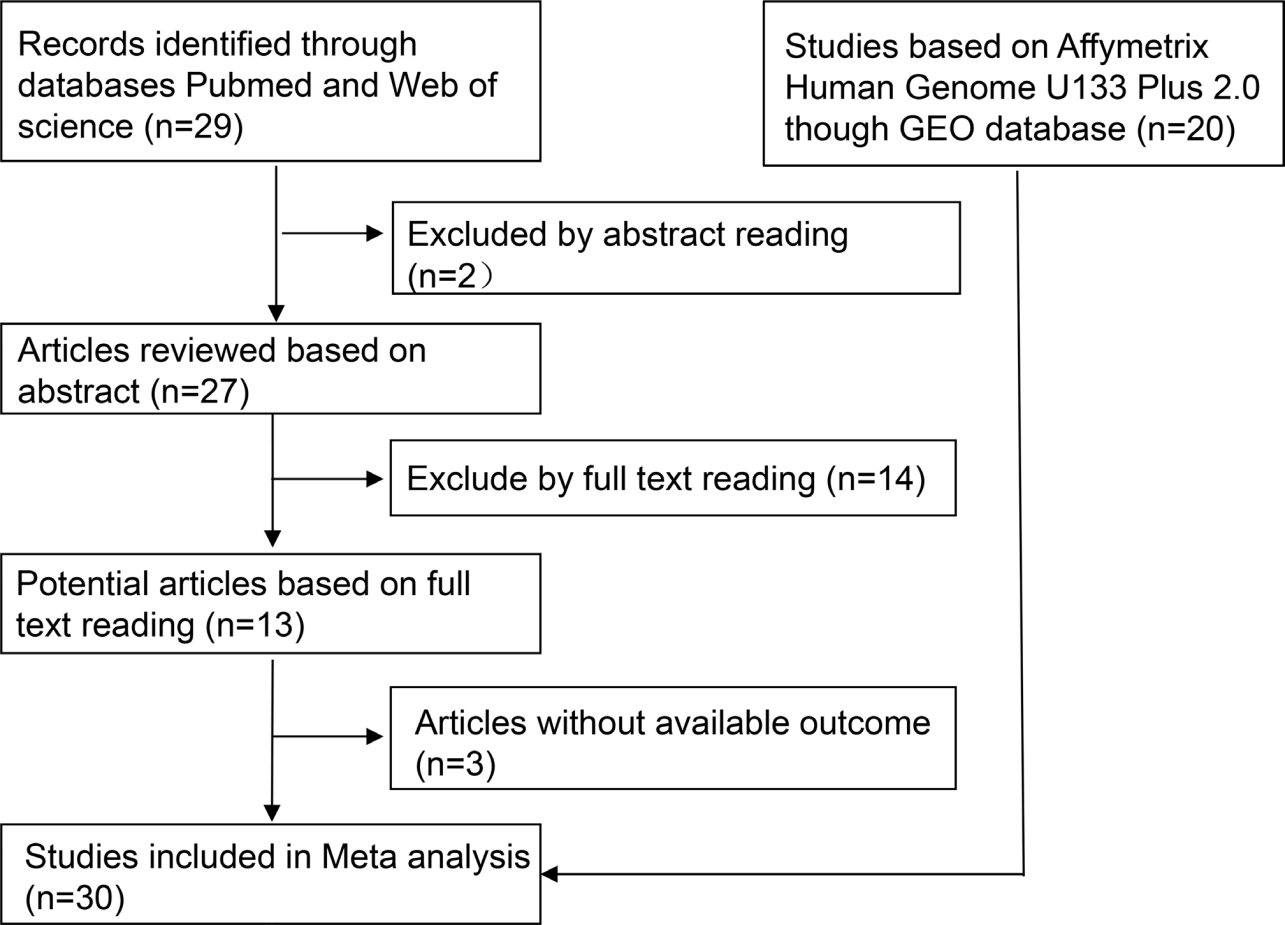
The flowchart of the meta-analysis Researchers acquired 20 RNA microarray datasets from the GEO database. Of the 29 publications identified in initial research, the authors selected 10 publications for further analysis by reading abstracts and full texts. Thus, there were 30 studies included in this meta-analysis.

As shown in Figure 1, we downloaded 20 Affymetrix Human Genome U133 Plus 2.0 datasets. We also found 10 relevant articles [14–21, 25–32]. Following the review process, 30 studies were chosen for the meta-analysis.

A total of 3482 patients were represented across these 30 studies, with a maximum sample size of 579 and a minimum sample size of 33. The mean sample size was 120. The most recent publication date was March 2017. The regions represented in the studies include China (9), America (9), Canada (5), Germany (2), Sweden (1), Japan (1), France (1), and Denmark (1). A total of 15 types of cancer were included in the meta-analysis (5 lung cancer, 4 breast cancer, 3 ovarian cancer, 3 DLBCL, 3 hepatocellular carcinoma, 1 colorectal cancer, 2 pancreatic cancer, 1 glioma, 1 nasopharyngeal carcinoma, 1 colon cancer, and 1 CN-AML).

All studies included a high AFAP1-AS1 expression group and a low AFAP1-AS1 expression group. 16 studies analyzed the expression level of AFAP1-AS1 by RT-PCR, while 19 utilized gene chips and 1 utilized fluorescence *in situ* hybridization (FISH). OS and RFS were estimated as a survival outcome in 76% (22) and 21% (6) of the studies, respectively. Associations between AFAP1-AS1 and clinical characteristics of cancers - TNM stage, lymph node metastasis, and distant metastasis - were estimated by 9, 5 and 4 studies, respectively.

### GEO data analysis

**Figure 2:**
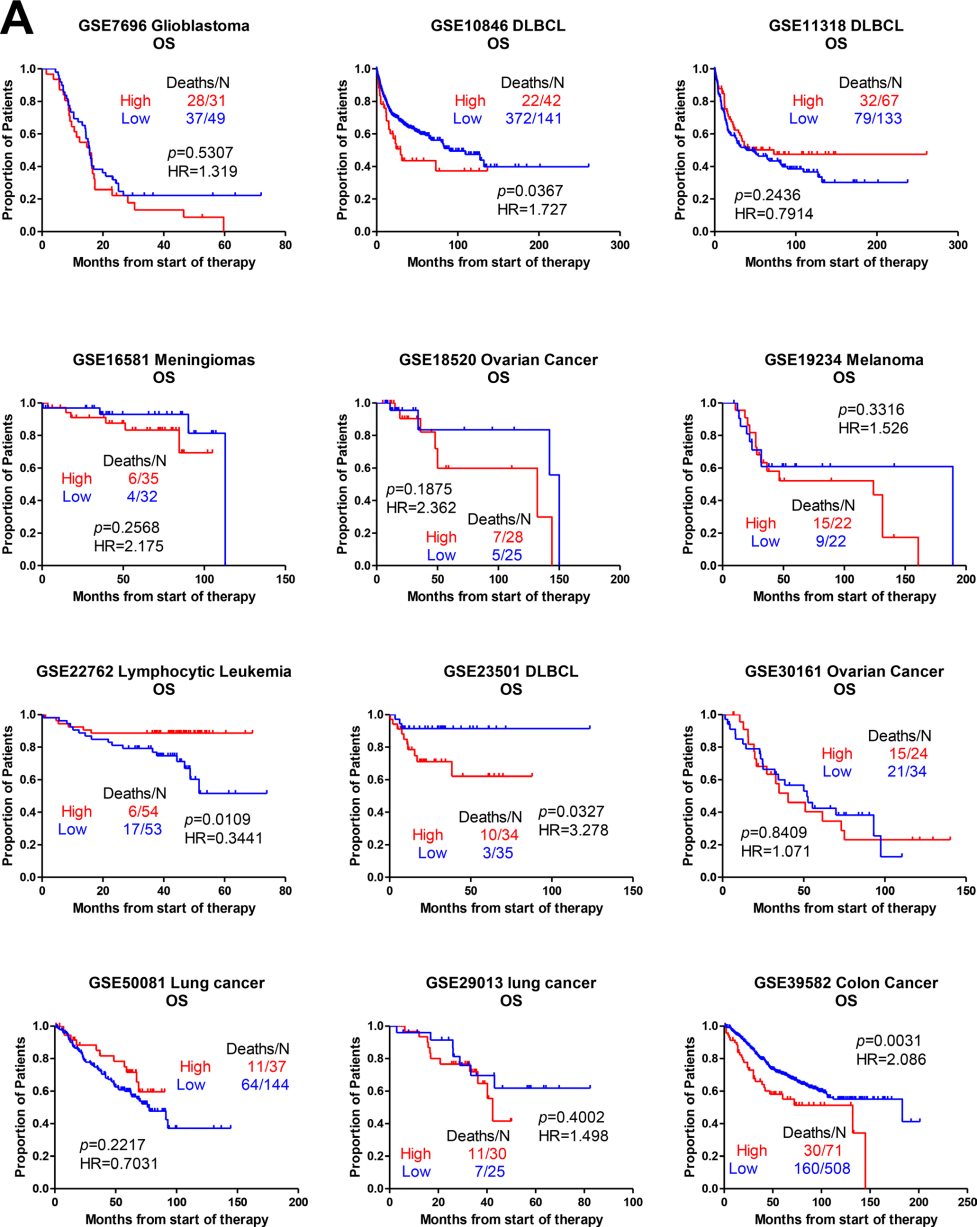

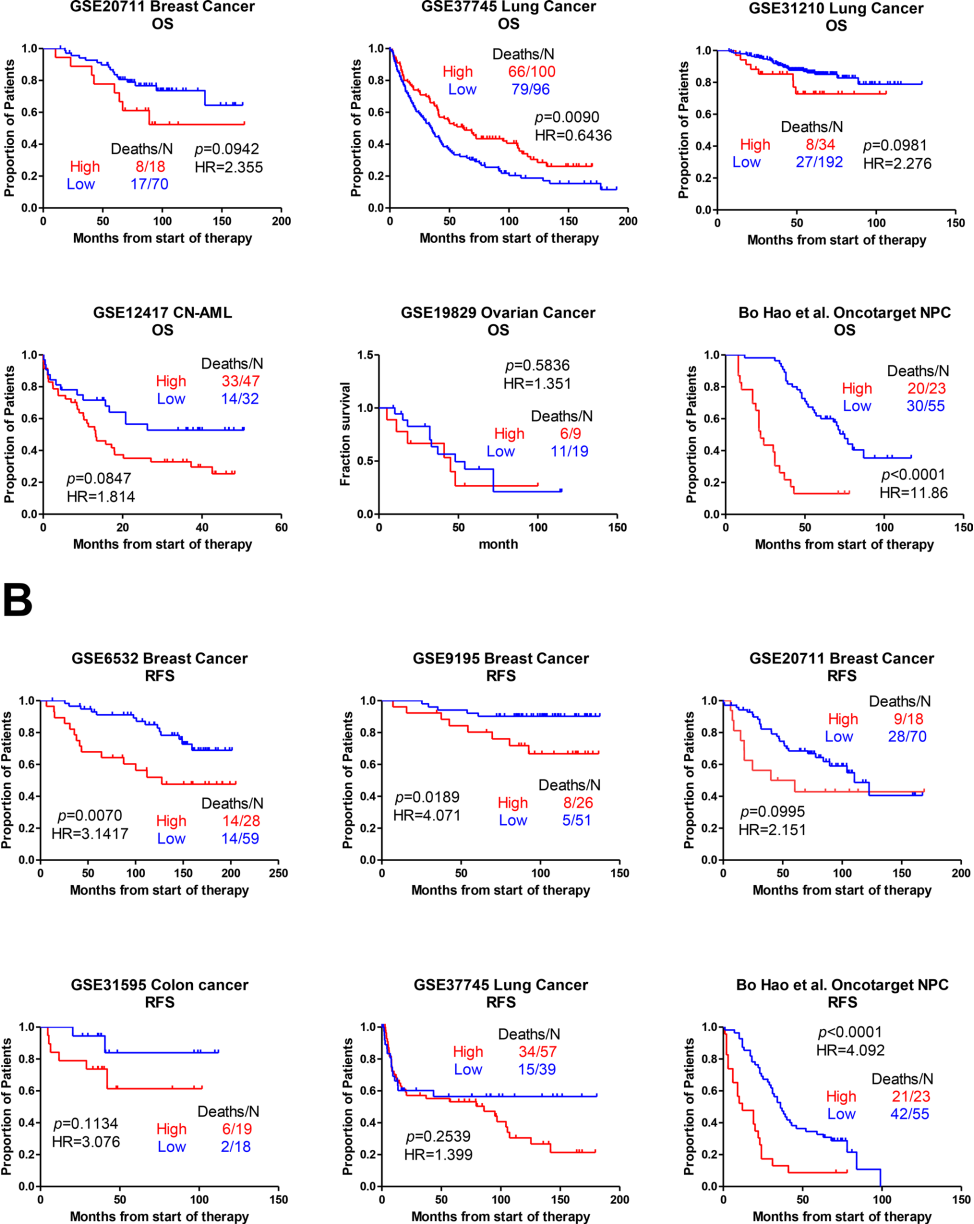
Kaplan-Meier curves relating AFAP1-AS1 expression to OS/RFS in different cancers The relationship between AFAP1-AS1 and survival (**A**) OS, and (**B**) RFS. HR and 95% CIs were calculated in different cancers. The cut-off value for differentially expressed lncRNA was set at ≥ 1.5-fold difference and the false discovery ratio (FDR) was < 0.05.

We analyzed the relationship between AFAP1-AS1 and survival (OS/RFS). The Kaplan-Meier curve (Figure 2), as well as the HR and 95% CI (Table 1), were derived. The cut-off value for differentially expressed lncRNA was set at ≥ 1.5-fold, and the false discovery ratio (FDR) was < 0.05. We calculated the HR between AFAP1-AS1 and clinical characteristics of cancers (i.e., TNM stage, lymph node metastasis and distant metastasis) (Table 2).

**Table 1:** Characteristics of articles included in the meta-analysis

Study	Year	Region	Tumor type	Sample size	Clinical stage of tumor	Test methods of AFAP1-AS1 expression	Survival outcome measures	Survival analysis	Reference
Zhou et al.	2016	China	Esophageal Cancer	162	TNM stage; distant metastasis; lymph node metastases	qRTPCR	OS	COX analysis	[28]
Zhang et al.	2016	China	Hepatocellular Carcinoma	78	TNM stage; lymph node metastases	qRTPCR	OS	COX analysis	[27]
Wang et al.	2016	China	Colorectal Cancer	52	TNM stage; distant metastasis	qRTPCR	OS	COX analysis	[25]
Lu et al.	2017	China	Cholangiocarcinoma	56	TNM stage; distant metastasis	qRTPCR	OS	K-M curve	[26]
Ma et al.	2016	China	Gallbladder Cancer	40	TNM stage; lymph node metastases	qRTPCR	None	None	[31]
Ye et al.	2016	China	Pancreatic Cancer	90	TNM stage; lymph node metastases	qRTPCR	OS	K-M curve	[21]
Deng et al.	2015	China	Lung Cancer	121	TNM stage; distant metastasis; lymph node metastases	qRTPCR	OS	COX analysis	[17]
Bo et al.	2015	China	Nasopharyngeal Carcinoma	112	TNM stage	ISH	OS RFS	COX	[30]
Luo et al.	2016	China	Esophageal Cancer	50	TNM stage	qRTPCR	None	None	[18]

**Table 2:** Survival characteristics of studies based on Affymetrix Human Genome U133 Plus 2.0

Type of cancer	GEO number	Region	No. of patients	Outcome measure	HR	*p* value
Lung Cancer	GSE31210	Japan	226	OS	2.276	0.0981
DLBCL	GSE11318	USA	200	OS	0.7914	0.2436
Glioblastoma	GSE7696	Canada	80	OS	1.319	0.5307
DLBCL	GSE10846	USA	414	OS	1.727	0.0367
Meningioma	GSE16581	USA	67	OS	2.175	0.2568
Ovarian Cancer	GSE18520	USA	53	OS	2.362	0.1875
Melanoma	GSE19234	USA	44	OS	1.526	0.3316
Ovarian Cancer	GSE19829	USA	28	OS	1.351	0.5836
Breast Cancer	GSE20711	Canada	88	OS	2.355	0.0942
Lymphocytic Leukemia	GSE22762	Germany	107	OS	0.344	0.0109
DLBCL	GSE23501	USA	69	OS	3.278	0.0327
Ovarian Cancer	GSE30161	USA	58	OS	1.071	0.8409
CN-AML	GSE12417	Germany	79	OS	1.814	0.0847
Lung Cancer	GSE37745	Sweden	196	OS	0.644	0.0090
Lung Cancer	GSE29013	USA	55	OS	1.498	0.4002
Colon Cancer	GSE39582	France	579	OS	2.086	0.0031
Lung Cancer	GSE50081	Canada	181	OS	0.703	0.2217
Breast Cancer	GSE6532	Canada	87	RFS	3.142	0.0070
Breast Cancer	GSE9195	Canada	77	RFS	4.071	0.0189
Breast Cancer	GSE20711	Canada	88	RFS	2.151	0.0995
Colon Cancer	GSE31595	Denmark	33	RFS	3.076	0.1134
Lung Cancer	GSE37745	Sweden	96	RFS	1.399	0.2539

### Association between AFAP1-AS1 and survival in 14 types of cancers

**Figure 3:**
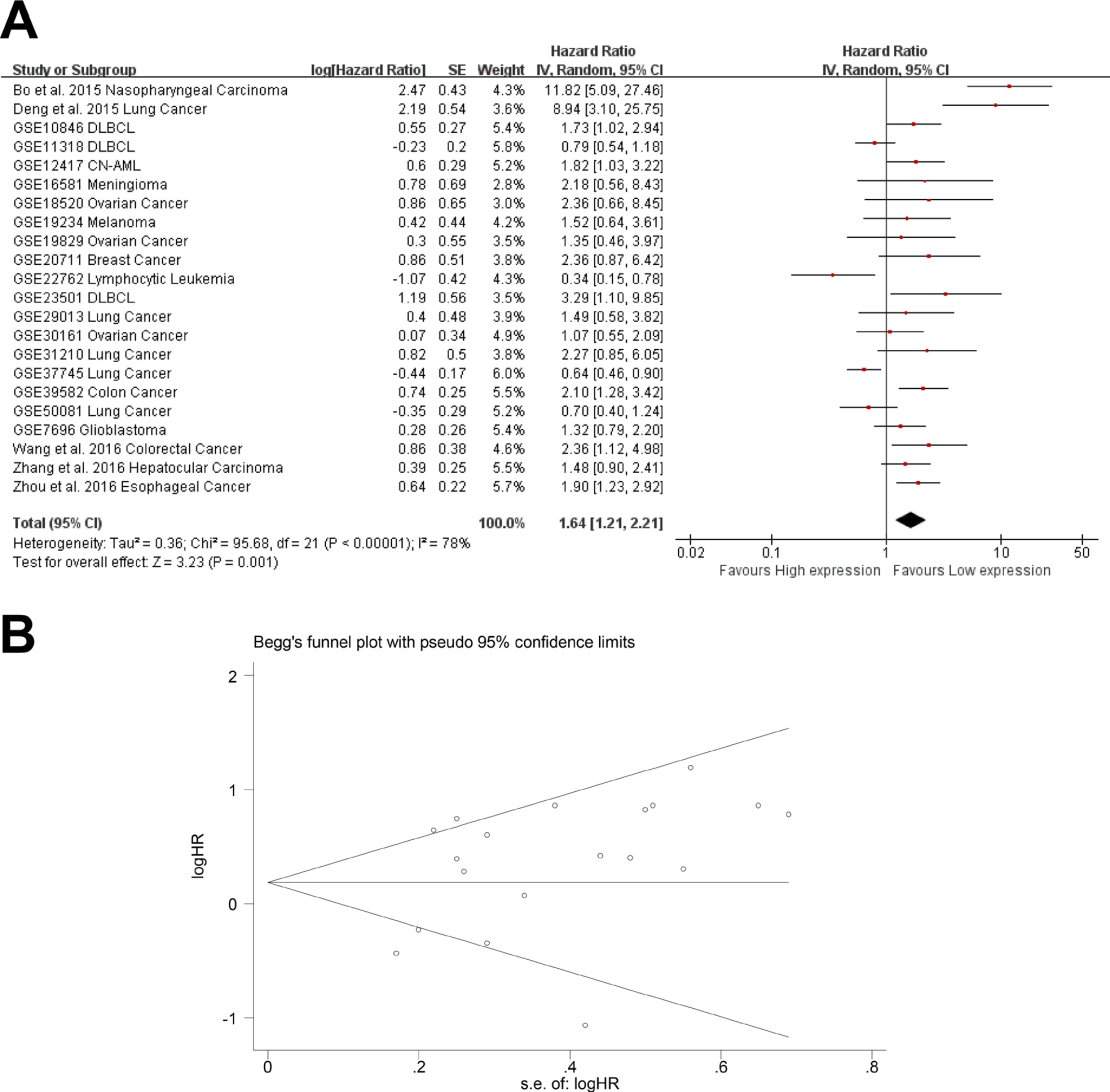
Meta-analysis of the independent role of AFAP1-AS1 in OS of different cancers (**A**) Twenty-two studies used meta-analysis to identify the pooled HR of OS (pooled HR: 1.58; 95% CI: 1.21-2.21); (**B**) There was no significant publication bias for OS (Egg's test *P* > |t| = 0.245 > 0.05, Begg's test Pr > |z| = 0.401)

In total, 22 studies used a Cox analysis to obtain the HR of OS. A significant association was found between AFAP1-AS1 and OS in cancer patients (pooled HR: 1.58; 95% CI: 1.21–2.21, Figure 3A). Significant heterogeneity existed between studies (Tau^2^ = 0.36; Chi^2^ = 95.68, df = 21 (*P* < 0.00001); I^2^ = 78%), and there was no significant publication bias (Egg's test *P* > |t| = 0.245 > 0.05, Begg's test Pr > |z| = 0.401) (Figure 3B). We used both the fixed effect model and random effect model and found that the results did not differ between the two models.

**Figure 4:**
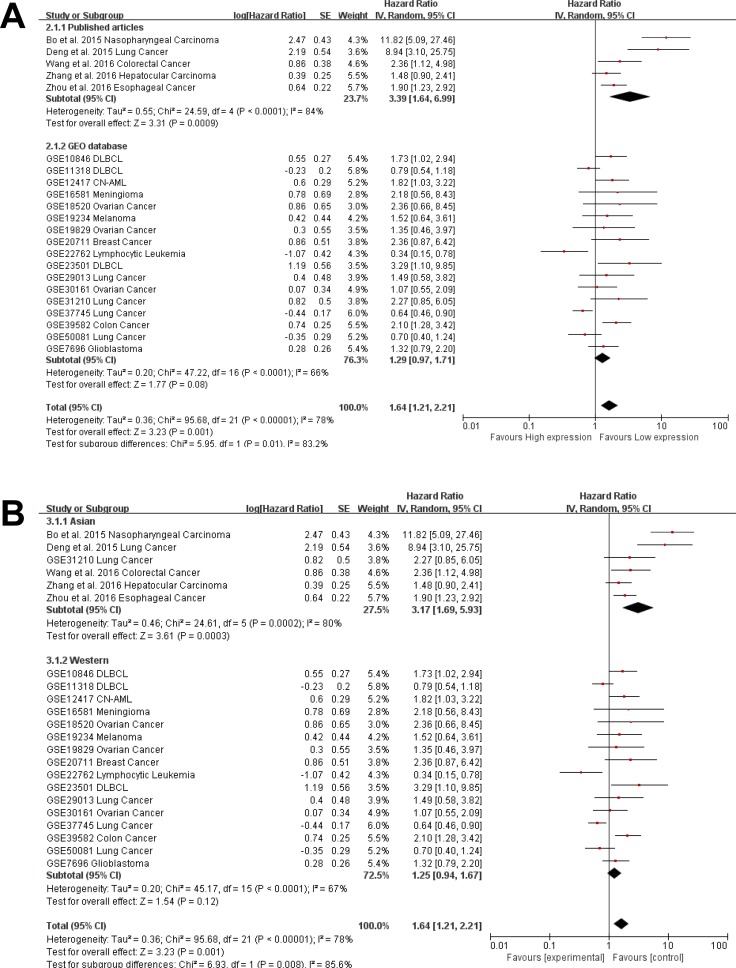
Subgroup analysis of independent regions and data sources in OS (**A**) Subgroups were analyzed for the presence of heterogeneity based on the data source. The association between AFAP1-AS1 and OS of cancer patients was significant in the Asian population (pooled HR: 3.17, 95% CI: 1.69–5.93), but not significant in the western population (pooled HR: 1.25, 95% CI: 0.94–6.99) but not in data from the GEO database (pooled HR: 1.29, 95% CI: 0.97–.71).

Due to the presence of heterogeneity, subgroups were analyzed for data source (Figure 4A) and region (Figure 4B). We found a significant association between AFAP1-AS1 and the OS of cancer patients in Asian countries (pooled HR: 3.17, 95% CI: 1.69–5.93). This association was not significant in western regions (pooled HR: 1.25, 95% CI: 0.94–1.67). AFAP1-AS1 was found to be significantly associated with the OS of cancer patients in data from published articles (pooled HR: 3.39, 95% CI: 1.64–6.99). The association was not significant for data from the GEO database (pooled HR: 1.29, 95% CI: 0.97–1.71). There was less significant heterogeneity across studies in the western subgroup (Tau^2^ = 0.20; Chi^2^ = 45.17, df = 15 (*P* < 0.0001); I^2^ = 67%) as well as studies from the GEO database (Tau^2^ = 0.20; Chi^2^ = 47.22, df = 16 (*P* < 0.0001); I^2^ = 66%). Greater heterogeneity existed in the Asian subgroup (Tau^2^ = 0.46; Chi^2^ = 24.61, df = 5 (*P* = 0.0002); I^2^ = 80%) and in the data collected from published articles (Tau^2^ = 0.55; Chi^2^ = 24.59, df = 4 (*P* < 0.0001); I^2^ = 84%).

**Figure 5:**
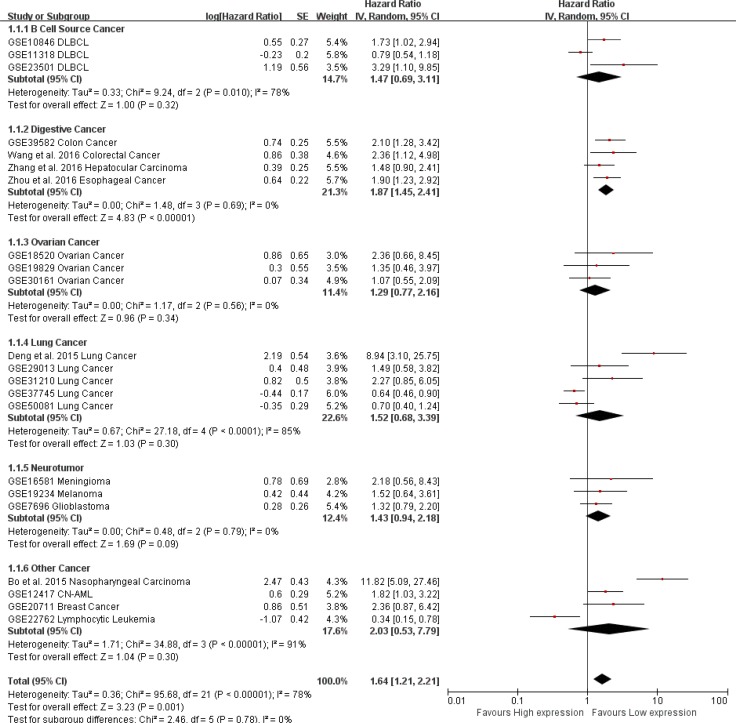
Subgroup analysis of tumor types in OS/RFS AFAP1-AS1 was a risk factor in the prognosis of lung cancer (pooled HR: 1.54; 95% CI: 1.01–2.34), digestive system cancer (pooled HR: 1.87; 95% CI: 1.45–2.41) and nasopharyngeal carcinoma (HR: 11.82; 95% CI: 5.09–27.46).

To maximize clinical relevance, subgroups were analyzed based on tumor type. We found that AFAP1-AS1 was a risk factor in the prognosis of lung cancer (pooled HR: 1.54; 95% CI: 1.01–2.34), digestive system cancer (pooled HR: 1.87; 95% CI: 1.45–2.41) and nasopharyngeal carcinoma (HR: 11.82; 95% CI: 5.09–27.46). However, there was no significant association between AFAP1-AS1 and OS in lung cancer, ovarian cancer, or tumors of the nervous system. This result was strengthened by the low heterogeneity between the studies (Figure 5).

**Figure 6:**
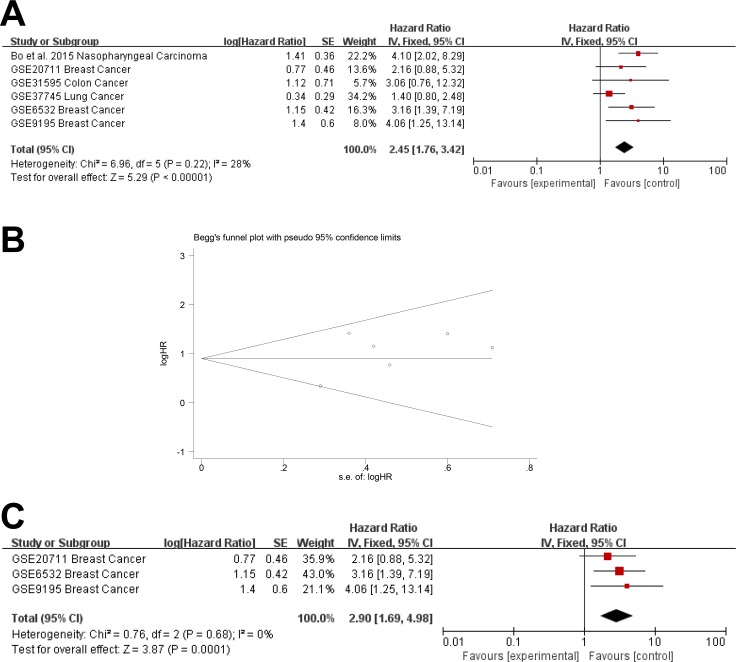
Meta-analysis of the independent role of AFAP1-AS1 in RFS of different cancers (**A**) Six studies used meta-analysis to identify the pooled HR for RFS (pooled HR: 2.45, 95% CI: 1.76-3.42). (**B**) There was no significant publication bias for RFS (Begg's test: Pr > |z| = 1.000, Egg's test: *P* > |t| = 0.271 > 0.05). (**C**) AFAP1-AS1 was a risk factor for RFS in breast cancer (pooled HR = 2.90; 95% CI: 1.69–4.98).

The prognostic value of AFAP1-AS1 in RFS was evaluated in 6 studies. AFAP1-AS1 was significantly associated with RFS (pooled HR: 2.45, 95% CI: 1.76–3.42) (Figure 6A). There was no significant heterogeneity across the studies (Heterogeneity: Chi^2^ = 6.96, df = 5 (*P* = 0.22); I^2^ = 28%), nor was there significant publication bias (Begg's test: Pr > |z| = 1.000, Egg's test: *P* > |t| = 0.271 > 0.05) (Figure 6B). Three studies focused on breast cancer and a subgroup analysis found that AFAP1-AS1 was significantly associated with RFS in breast cancer (pooled HR = 2.90; 95% CI: 1.69–4.98; heterogeneity: Chi^2^ = 0.76, df = 2 (*P* = 0.68); I^2^ = 0%) (Figure 6C). Both fixed effect and random effect models were used to calculate the effect and the results were not markedly different between the two models.

### Association between AFAP1-AS1 and the clinical characteristics of cancers

As shown in Table 3, 10 studies examined the association between AFAP1-AS1 and clinical characteristics in 6 types of cancer. 9 studies examined the association between TNM stage and AFAP1-AS1 in different cancers, including esophageal cancer (2), liver cancer (3), pancreatic cancer (1), lung cancer (2) and colorectal cancer (1). There was a significant association in esophageal cancer (pooled OR = 1.90; 95% CI: 1.01–3.57) and colorectal cancer (OR = 6.72; 95% CI: 1.92–23.58) but no significant association in lung cancer (pooled OR = 2.84; 95% CI: 0.12–65.10) and pancreatic cancer (OR = 2.15; 95% CI: 0.90–5.15). Association results for the two lung cancer studies were contradictory (Fixed effect pooled OR = 2.11; 95% CI: 1.25–3.55; Random effect pooled OR = 2.11; 95% CI: 0.78–5.66). There were 5 studies examining lymph node metastases in lung cancer (1), pancreatic cancer (1), liver cancer (1), esophageal cancer (1), and gallbladder cancer (1). We observed a significant association in lung cancer (OR = 2.85; 95% CI: 1.35–6.04), liver cancer (OR = 4.10; 95% CI: 1.43–11.75), pancreatic cancer (OR = 5.25; 95% CI: 2.09–13.20), and esophageal cancer (OR = 3.24; 95% CI: 1.70–6.17) but no significant association in gallbladder cancer (OR = 1.56; 95% CI: 0.44–5.53). There were 4 studies that examined distant metastases in different cancers, including lung cancer (1), liver cancer (1), colorectal cancer (1), and esophageal cancer (1). AFAP1-AS1 was significantly associated with distant metastasis in lung cancer (OR = 2.24; 95% CI: 1.08–4.67), colorectal cancer (OR = 7.50; 95% CI: 2.01–28.05), and esophageal cancer (OR = 2.98; 95% CI: 1.28–6.97) but not in liver cancer (OR = 3.33; 95% CI: 0.78–14.23). Subgroup analysis, sensitivity analysis and appraisal of publication bias were not performed due to the limited number and relative homogeneity of the studies.

**Table 3:** Results of meta-analysis of increased AFAP1-AS1 expression and clinical features in various cancers

Cancer types	No. of studies	No. of patients	Pooled OR		Heterogeneity
			Fixed	Random	I^2^
**TNM stage**					
Liver Cancer	3	290	2.11 [1.25, 3.55]	2.11 [0.78, 5.66]	68%
Esophageal Cancer	2	232	1.90 [1.01, 3.57]	1.89 [1.01, 3.56]	0%
Pancreatic Cancer	1	90	2.15 [0.90, 5.15]	2.15 [0.90, 5.15]	Not applicable
Colorectal Cancer	1	52	6.72 [1.92, 23.58]	6.72 [1.92, 23.58]	Not applicable
Lung Cancer	2	317	1.75 [0.98, 3.15]	2.84 [0.12, 65.10]	93%
			2.11 [1.56, 2.84]	2.30 [1.30, 4.09]	68%
**Lymph node metastasis**					
Gallbladder Cancer	1	50	1.56 [0.44, 5.53]	1.56 [0.44, 5.53]	Not applicable
Pancreatic Cancer	1	90	5.25 [2.09, 13.20]	5.25 [2.09, 13.20]	Not applicable
Liver Cancer	1	78	4.10 [1.43, 11.75]	4.10 [1.43, 11.75]	Not applicable
Esophageal Cancer	1	162	3.24 [1.70, 6.17]	3.24 [1.70, 6.17]	Not applicable
Lung Cancer	1	121	2.85 [1.35, 6.04]	2.85 [1.35, 6.04]	Not applicable
			3.28 [2.24, 4.79]	3.29 [2.24, 4.81]	0%
**Distant metastasis**					
Lung Cancer	1	121	2.24 [1.08, 4.67]	2.24 [1.08, 4.67]	Not applicable
Liver Cancer	1	56	3.33 [0.78, 14.23]	3.33 [0.78, 14.23]	Not applicable
Colorectal Cancer	1	52	7.50 [2.01, 28.05]	7.50 [2.01, 28.05]	Not applicable
Esophageal Cancer	1	162	2.98 [1.28, 6.97]	2.98 [1.28, 6.97]	Not applicable
			3.05 [1.89, 4.92]	3.02 [1.86, 4.89]	0%

## DISCUSSION

**Figure 7:**
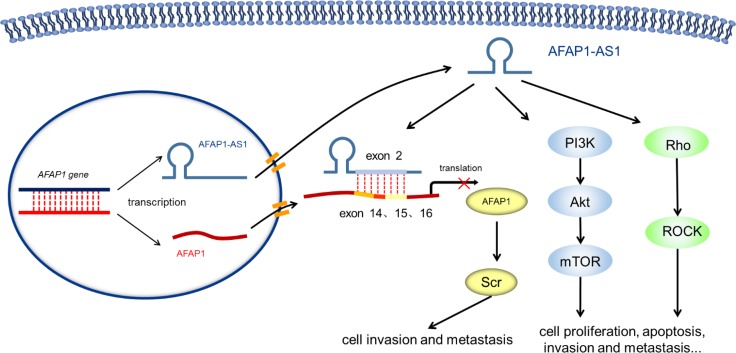
Molecular mechanisms of AFAP-AS1 in human cancer AFAP1-AS1 is involved in tumorigenesis and development through the regulation of various pathways including Rho/Rac and PI3K-Akt. On the other hand, as the antisense lncRNA of AFAP1, the second exon of AFAP1-AS1 is complementary to exons 14, 15, and 16 of AFAP1. AFAP1-AS1 could inhibit AFAP1 protein translation, thus affecting cell motility, tumor invasion, and metastasis.

The expression of the lncRNA AFAP1-AS1 has been shown by ourselves and others to be significantly upregulated in various tumor types. Additionally, high AFAP1-AS1 expression is closely correlated to a poor prognosis of cancer patients [16, 21, 27, 28, 30, 32]. *In vitro* experiments have shown that tumor cell characteristics, including proliferation and metastasis, can be inhibited by down-regulating AFAP1-AS1 expression. Further mechanistic studies found that AFAP1-AS1 was involved in tumorigenesis and development through the regulation of pathways including Rho/Rac [30] and PI3K-Akt [20]. On the other hand, as the antisense lncRNA of AFAP1, the second exon of AFAP1-AS1 is complementary to exons 14, 15, and 16 of AFAP1. Our group was the first to demonstrate that the specific knock-down of AFAP1-AS1 resulted in AFAP1 expression inhibition [30]. This lncRNA may be involved in the regulation of related proteins such as Src, thus affecting the cell motility, tumor invasion, and metastasis. (Figure 7)

In our meta-analysis, we identified high expression of AFAP1-AS1 in various cancers, including 22 studies with OS data. By pooled HR values, AFAP1-AS1 was found to be an independent risk factor (pooled HR = 1.58; 95% CI: 1.21–2.21) of OS in tumor patients. In addition, 6 studies analyzed RFS and demonstrated that AFAP1-AS1 could be regarded as an independent prognostic biomarker of RFS (pooled HR = 2.45, 95% CI: 1.76–3.42).

Three previous studies found that high expression of AFAP1-AS1 correlated with TNM staging (III/IV vs. I/II) in esophageal cancer and colorectal cancer. Our study, which is based on 981 patients from five studies with TNM staging data, indicated that the correlation between high expression of AFAP1-AS1 and tumor TNM staging has a pooled OR value of 2.30 (95% CI: 1.30–4.09). However, this correlation may be possible in unstudied tumor types.

The correlation between AFAP1-AS1 expression and lymph node metastasis and distant metastasis was analyzed in five and four studies, respectively. AFAP1-AS1 was significantly associated with distant metastasis in lung cancer, colorectal cancer, and esophageal cancer but not in liver cancer. Additionally, AFAP1-AS1 was significantly associated with lymphocyte metastasis in lung cancer, liver cancer, pancreatic cancer, and esophageal cancer but not in gallbladder cancer. The pooled ORs (lymphocyte metastasis: OR = 3.28, 95% CI: 2.24–4.79, distant metastasis: HR = 3.05, 95% CI: 1.89–4.92) confirm the correlation between AFAP1-AS1 expression and lymphocyte metastasis and distant metastasis. Nevertheless, considering the inadequate sample size, our conclusions could change with the inclusion of more data.

In addition, our group recently found that AFAP1-AS1 expression was related to PD1 expression, indicating the possible participation of AFAP1-AS1 in the fascinating field of tumor immunology [15]. As the current star molecule in the field of tumor immunology, PD1 plays an important role in the immune tolerance of tumor cells [5, 33]. At present, anti-PD1 drugs (represented by Keytruda and Opdivo) have proven to be effective tumor therapies [34–38]. The close correlation between AFAP1-AS1 and PD1 indicates the potential of AFAP1-AS1 as a future target for immunological therapies and provides new avenues for treatment [39–41].

Nevertheless, there are many deficiencies in this meta-analysis. (1) Only English-language publications were included in this study, so any data demonstrating a correlation between AFAP1-AS1 and tumor prognosis in other languages were ignored. (2) Publications with positive results were more likely to get published than those with negative results, so data from negative as well as ongoing studies were underrepresented. Thus, the role of AFAP1-AS1 in tumor prognosis may be over-estimated. (3) The degree of AFAP1-AS1 expression varied between different publications, making a uniform analysis difficult. (4) Survival time was largely related to the therapeutic regimen, thus differences in treatment regimens influenced the calculation of HR values as well as study heterogeneity. (5) The variance in publication quality was one of the main factors influencing the analysis. (6) The differences between cancer sub-types were not considered in this analysis and were also rarely reported in most publications.

In conclusion, AFAP1-AS1 can act as a newfound independent biomarker for predicting cancer prognosis. However, more evidence is necessary to demonstrate the correlation between high AFAP1-AS1 expression levels and clinical indices such as TNM staging, lymphocyte metastasis, distant metastasis, and histological stage. Moreover, most patients in our study were Asian, thus our results do not reflect the global situation. Great heterogeneity remained in the study of the correlation between AFAP1-AS1 and clinical diagnosis. This can be explained by the application of different methods to classify high or low expression of AFAP1-AS1. Therefore, the conclusions from our study need to be further confirmed through multi-center randomized controlled trials with large sample sizes.

## CONCLUSIONS

AFAP1-AS1 can serve as a novel molecular marker in different types of cancers. Further studies are necessary to demonstrate the correlation between high AFAP1-AS1 expression and clinical characteristics in order to improve the potential clinical benefits.

## Abbreviations

lncRNAs: long noncoding RNAs
AFAP1-AS1: AFAP1 antisense RNA 1
HR: hazard ratio
OR: odds ratio
CI: Confidence interval
AFAP1: actin filament associated protein 1
OS: overall survival
RFS: recurrence-free survival
FISH: fluorescence *in situ* hybridization
Rho: rhodopsin
PI3K: phosphoinositide-3-kinase
